# EHMTI-0101. Is inflammation atherogenic in neurological diseases? A case-control study with migraine and multiple sclerosis patients

**DOI:** 10.1186/1129-2377-15-S1-F20

**Published:** 2014-09-18

**Authors:** VG Quintanilla, M Toriello, J Castillo, J Fernandez, R Martinez-Nieto, S Montes, E Pons, S Gutierrez, E Palacio, A Oterino

**Affiliations:** 1 Neurology, Hospital Universitario Marqués de Valdecilla, Santander, Spain; 2 General Practitioner, Camargo Costa Health Center, Camargo (Cantabria), Spain; 3 General Practitioner, Camargo Costa Health Center, Santander, Spain; 4 Technician, Hospital Universitario Marqués de Valdecilla, Santander, Spain; 5 Nurse, Hospital Universitario Marqués de Valdecilla, Santander, Spain

## Introduction

There has been some debate concerning the endothelial damage and cerebrovascular risk in migraine.

## Aim

We will evaluate the endothelial damage in migraine comparing carotid intima-media thickness (IMT) and the endothelial-dependant flow-mediated vasodilation (EDV) with healthy controls and an active group, such as multiple sclerosis (MS).

## Methods

Subjects were matched for sex and age (range 20-55). McDonnald's 2010 criteria were used for MS; IHS-2004 and 2006 criteria for episodic migraine (EM) and chronic migraine (CM). IMT, EDV, and other vascular parameters, were obtained by a certified blind examiner. NO, von Willebrand factor (vWF), ICAM-1 and VCAM-1 were determined. Statitics Student's t test, general lineal models with post-hoc Bonferroni correction with adjusted means, and Pearson regression test.

## Results

We recruited 22 controls, 59 migraine patients (25 CM), 33 MS patients. IMT was thicker in MS than in controls (p=5.4E-009), EM (p=8.9E-006), and CM patients (p=0.008). CM had thicker IMTs than controls (p=0.001). IMT correlated with EDSS (r=0.464; p=0.011), and inversely with EDV (r=-0.414; p=0.000013) and BHI (r=-0.300; p=0.015). BHI inversely correlated with vWF (r=-0.317; p=0.011). EDV, was higher in controls than in MS (p=5.6E-006), and CM (p=0.001). MS and CM still predicted IMT and EDV under the model corrected for age and BMI (p<0.001).

## Conclusion

Our findings suggest intrinsic endothelial vascular damage which was found more consistently for MS than in CM patients. We hypothesize that endothelial damage could be associated to the neuroinflammation status itself.

Funded by 'Fundación Salud 2000', Merck, Juste, Novartis and FISS PI11/1232 grants.

